# Supplementary material for: Novel QTL Associated with Aerenchyma-Mediated Radial Oxygen Loss (ROL) in Rice (Oryza sativa L.) under Iron (II) Sulfide
Source: Plants (Basel). 2022 Mar 16;11(6):788. doi: 10.3390/plants11060788 (PMC8948734; doi:10.3390/plants11060788)
Supplement: Supplementary file 1 [file plants-11-00788-s001.zip › plants-1539996-supplementary.pdf]

plants

MDPI

Novel QTL Associated with Aerenchyma-Mediated Radial Oxygen Loss (ROL) in Rice (*Oryza sativa* L.) under Iron (II) Sulfide

Dang Van Duyen [2,†], Yongho Kwon [1,†], Nkulu Rolly Kabange [1,†,*], Ji-Yoon Lee [1], So-Myeong Lee [1], Ju-Won Kang [1], Hyeonjin Park [1], Jin-Kyung Cha [1], Jun-Hyeon Cho [1], Dongjin Shin [1] and Jong-Hee Lee [1,*]

[1] Department of Southern Area Crop Science, National Institute of Crop Science, RDA, Miryang, 50424, Korea; Y.H.K., kwon6344@korea.kr; J.-Y.L., minitia@korea.kr; S.M.L., olivetti90@korea.kr; J.-W.K., kangjw81@korea.kr; H.P., tinapark@korea.kr; J.K.C., jknzz5@korea.kr; J-H.C., hy4779@korea.kr; and D. J. S., jacob1223@korea.kr

[2] Molecular Biology Department, Agricultural Genetic Institute, Hanoi 11917, Vietnam; dangvanduyen79@gmail.com

* Correspondence: N.R.K., rollykabange@korea.kr, Tel.: +8210-5413-4881; ccriljh@korea.kr, Tel: +82–53–350–1169, Fax: +82–55–352–3059

† These authors contributed equally to this work.

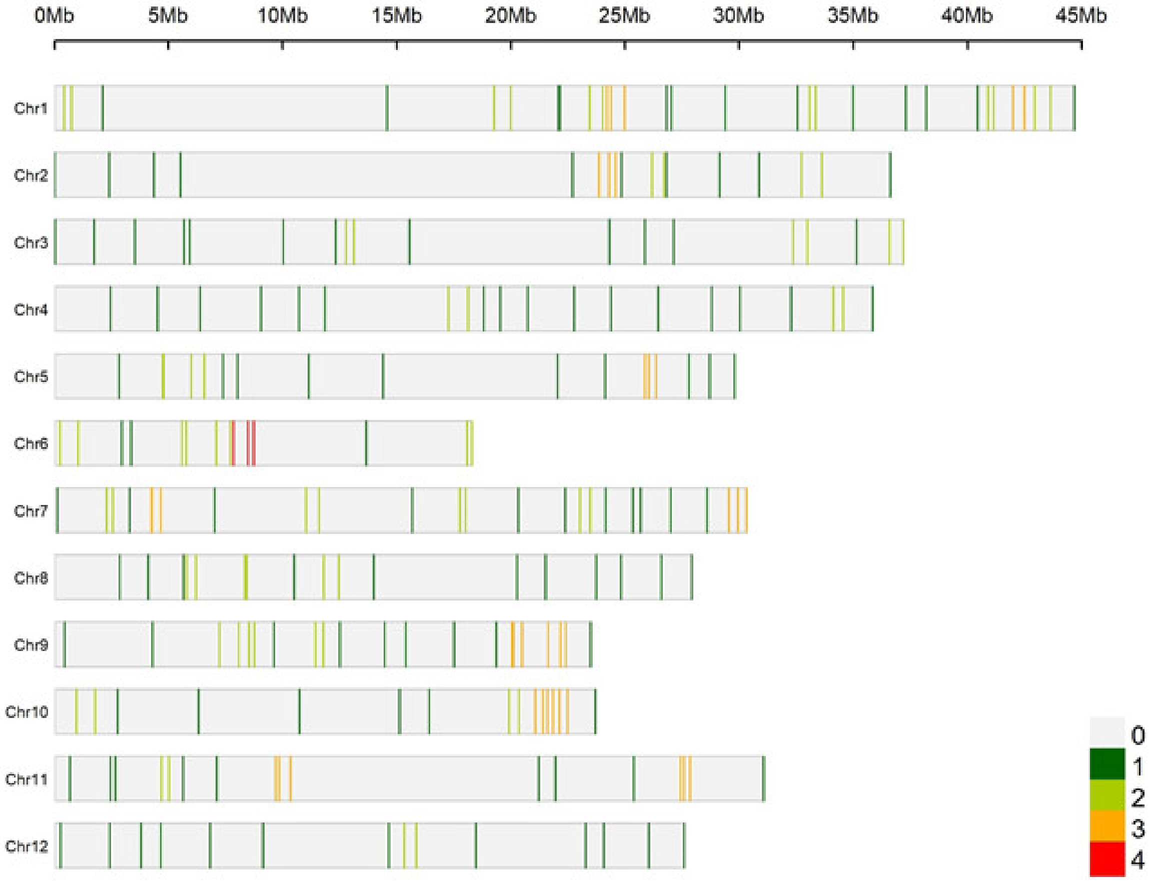

Figure S1. Density of Kompetitive Allele-Specific PCR (KASP) and Fluidigm markers across the rice genome.

Table S1. Results of Statistical Analysis of Traits in DH population

Trait	Sample Size	Mean	Variance	SE	Skewness	Kurtosis	Min	Max	W-test
ROL	117	27.79	55.99	7.481	0.2001	-0.2857	12.1	49	0.9791
RA	117	8.35	3.20	1.789	0.3287	-0.1947	4.283	13	0.9771
RL	117	6.71	11.18	1.057	-0.9184	0.8985	3.25	8.89	0.9407

ROL: radial oxygen loss; RA: root area; and RL: root length. W-test: the Shapiro–Wilk W-statistic for the test of normality of the distribution.

Table S2. Linkage groups and total length per chromosome

Chromosome ID	Chromosome Name	No. of markers	Length (cM)
1	Chromosome 1	29	207.95
2	Chromosome 2	17	189.28
3	Chromosome 3	20	204.8
4	Chromosome 4	21	201.48
5	Chromosome 5	17	139.66
6	Chromosome 6	11	60.54
7	Chromosome 7	27	210.84
8	Chromosome 8	17	102.5
9	Chromosome 9	19	101.21
10	Chromosome 10	13	109.7
11	Chromosome 11	16	130.12
12	Chromosome 12	13	134.52
Whole Genome	Whole Genome	220	1792.6

Table S3. List of ROL candidate genes and primers sequences used for qPCR validation.

Gene Name	Locus ID	Forward primer (5′->3′)	Reverse primer (5′->3′)	Tm °C (F/R)	G-C contents % (F/R)	Amplicon size (bp)
OsTCP7	LOC_Os02g42380	CCATGTCTACCAGCCCGTG	TTGCTGTGCCGGTCCTTC	60.15/60.28	63.16/61.11	150
OsTRX	LOC_Os02g42700	GCGGGCCATGCAAAATGATA	TGTCGGGGTTTTCATCAGTGT	59.61/59.86	50.00/47.62	102
OsMYB21	LOC_Os02g42850	GGTGGATCAACTACCTGCGG	TCCTAGACCACTTGTTGCCG	60.46/59.68	60.00/55.00	108
OsPLIM2a	LOC_Os02g42820	GGGCAGCTACAACCACTTGA	TCCTGTTCTTTCTCGCTGGC	60.25/60.32	55.00/55.00	102
OsDEF7	LOC_Os02g41904	CCACAGGTTCAAGGGCATGT	TTCTTGCAGAAGCACTTGCG	60.54/59.69	55.00/50.00	120
OsARF8	LOC_Os02g41800	CAGTCGAGCCTCATGTACCC	CCCATTGTCAGGTCAGTGCT	59.90/59.96	60.00/55.00	95
OsEXPA	LOC_Os02g42650	AATTCGAGGATGGAGACGGC	CCCCCAGATGTGCTTCATGT	59.89/60.03	55.00/55.00	89
OsNIP2	LOC_Os02g41860	GTGATCGGGTACAAGCACCA	ATGAAGATCATGCCGCCGAA	60.04/60.18	55.00/50.00	110
Oscb5	LOC_Os02g42740	GTAAAAGGCGACCGACAGGA	CCTGGGAAAACGTCGTCAGA	60.04/59.97	55.00/55.00	88
OsLRR2	LOC_Os02g42412	CAGCAGCTGTGTTAGGATCAC	GAGGGCATGACCGAACATCT	58.99/59.82	52.38/55.00	92
OsWBC8	LOC_Os02g41920	TCACAGTGGTGGTGTACTGG	ACCCGACGTATCTCTTCACC	59.24/58.90	55.00/55.00	126
OsActin1	LOC_ Os05g36290	CTAGCGGTCGAACAACTGGT	ACCGGAGGATAGCATGAGGA	57.5/57.5	55.00/55.00	102
